# A Comparison of the QUECHERSER Mega-Method for Pesticide Determination in Loamy-Clayed Soil and the Effect of Organic Amendments on Pendimethalin, Oxyfluorfen, and Trifloxystrobin Soil Persistence

**DOI:** 10.3390/jox15040098

**Published:** 2025-06-26

**Authors:** Rafael Boluda, Alejandro Alejos-Campo, Eva Fernández-Gómez, Miguel Gamón, Luis Roca-Pérez, Oscar Andreu-Sánchez

**Affiliations:** Departamento de Biologia Vegetal, Universitat de València, Av. Vicent Andrés i Estellés s/n, 46100 Burjassot, València, Spain; alejandro.alejos@uv.es (A.A.-C.); eva.fernandez-gomez@uv.es (E.F.-G.); gamonmig@gmail.com (M.G.); luis.roca@uv.es (L.R.-P.); oscar.andreu@uv.es (O.A.-S.)

**Keywords:** pesticide extraction, QuEChERS, QuEChERSER, persistence, degradation, organic amendment

## Abstract

The intensive use of pesticides has raised environmental concerns due to their persistence and slow degradation, posing ecotoxicological risks. Despite regulatory measures, pesticide application remains high, leading to soil and water contamination. To effectively monitor and mitigate these impacts, selecting an appropriate and efficient extraction method for detecting pesticides in soil is critical. This study evaluated the effectiveness of two extraction methods in soil—QuEChERS and QuEChERSER—and assessed the persistence of three commonly used pesticides. A test was conducted using 13 pesticide standards, representing a wide variety of functional groups, to compare the two extraction methods. For the persistence study, a microcosm experiment was performed with three selected pesticides: pendimethalin, oxyfluorfen, and trifloxystrobin. These were chosen due to their agricultural relevance, potential human toxicity, and persistence in various environmental compartments. The impact of two organic amendments on their dissipation was also evaluated. The microcosms were incubated in dark chambers at room temperature for 21 days, and pesticide concentrations were analyzed using ultra-high-performance liquid chromatography–tandem mass spectrometry. Both methods were effective, though performance varied depending on the compound. QuEChERSER proved to be more efficient, requiring less time and fewer resources than the traditional QuEChERS method. Among the three pesticides tested, the herbicide oxyfluorfen was the most persistent, while the fungicide trifloxystrobin showed the least persistence. The application of organic amendments enhanced the dissipation of all three pesticides. These findings highlight the importance of selecting appropriate extraction techniques and adopting sustainable agricultural practices to mitigate pesticide residues in the environment.

## 1. Introduction

Since the beginning of modern agriculture, the use of pesticides has become the norm for pest elimination. Despite the multiple advantages they offer, there are several problems posed by their use. Many of these substances have high mobility, so the vast majority end up elsewhere [1,2]. Another main characteristic of pesticides that threatens the environment is their persistence [3,4]. Most pass into the soil or volatilize and, depending on their characteristics, can reach groundwater or other environmental compartments, remaining there for a long time [5,6]. The long-term behavior of pesticides depends on soil properties, compound characteristics, and environmental factors, leading to volatilization, degradation, leaching, or accumulation [7]. Moreover, pesticides can negatively affect soil microbiota, disrupting microbial communities essential for soil health and nutrient cycling [8]. Some pesticides, such as those with xenoestrogenic activity, have been associated with risks to human health, including potential carcinogenic effects and endocrine disruptions, which increases the urgency for their control and monitoring [9].

One of the fundamental aspects in the determination of xenobiotics in soils is the choice of an appropriate extraction method, due to the structural and compositional complexity of soils, which often contain organic matter, clay minerals, and microbial communities that can interfere with analyte recovery [10,11,12]. The extraction of pesticides in different environment matrices has traditionally been carried out using the QuEChERS method known by its acronym (quick, easy, cheap, effective, rugged, and safe) [13], which has been used by numerous authors for the quantification of many contaminants in various matrices, Ex. [14,15,16,17]. Modifications of this method for contaminant extraction in a wide variety of matrices are continuously being validated [18,19,20,21,22]. Furthermore, in 2021, a new QuEChERS mega-method was published by one of the authors of the QuEChERS method: QuEChERSER (more than QuEChERS, adding “Efficient and Robust” to its acronym) [23], which captures a wider polarity range than QuEChERS and has already been validated in a variety of matrices for environmental contaminants, veterinary drugs, and pesticides [24,25,26]. However, at the time of writing this article, we have not found any reference to its application in soils. The study of the persistence of xenobiotics in soils and their interactions with the constituents of the edaphic environment continue to be topics of great interest and relevance [5,27,28,29] since they can be retained in the soil exchange complex, in organic matter, in the clay fraction or precipitates in the soil matrix, which can decrease their ecosystem functions and present risks to the health of plants, animals and humans [30,31,32].

Three widely used pesticides today—pendimethalin (PMDT) and oxyfluorfen (OXF), both herbicides, and the fungicide trifloxystrobin (TFXT) [33]—were selected for this study based on their extensive agricultural use, along with their documented toxicity and persistence in the environment. PMDT or 3,4-dimethyl-2,6-dinitro-N-pentan-3-ylaniline (CAS: 40487-42-1) is an herbicide that belongs to the group of dinitroanilines; it has a wide spectrum of application and is used in pre- and post-emergence grass and weed control in crops. It acts by inhibiting cell division and elongation [34]. It is considered to be irritating to the skin, eyes, and respiratory tract [35]. It is also considered to have negative effects on reproduction, and some studies suggest that it is associated with a higher incidence of pancreatic cancer [36]. Although it is not classified as persistent, its half-lives range from 76 to 98 days, which is relatively high compared to other compounds [37,38]. OXF or 2-chloro-1-(3-ethoxy-4-nitrophenoxy)-4-(trifluoromethyl) benzene (CAS: 42874-03-3) belongs to the chemical group of diphenyl ethers. It is a broad-spectrum herbicide used in both pre-emergence and post-emergence weed control. It is considered to be a persistent and immobile pesticide in soil since it binds to soil organic carbon [39]. In humans, its main toxic effects include liver dysfunction and impaired red blood cell production, which may lead to anemia [40]. TFXT, according to IUPAC: (2E)-2-methoxyimino-2-[2-[[(E)-1-[3-(trifluoromethyl) phenyl] ethylideneamino] oxymethyl] phenyl] acetate (CAS: 141517-21-7), is a fungicide of the strobilurin group, which acts by externally inhibiting quinone in complex 3 of the respiratory chain [41]. It is a foliar-applied compound for the control of certain foliar, stem, and root diseases. It is a widely used broad-spectrum fungicide, with very low water solubility and volatility [42]. It has low toxicity in mammals, but there is evidence that it is toxic to birds, fish, and aquatic invertebrates, causing negative effects on their fertility [43]. In some cases, it has been observed to decompose rapidly in soils, and its residues have been shown to dissipate below the detection limit in 15 days [44].

Protecting and preserving soil health is essential for the maintenance of its ecosystem functions. Its main functions are climate control, water quality and quantity maintenance, nutrient cycling, purification capacity, and providing habitat for a vast array of biodiversity [45,46]. As indicated above, the use of pesticides can inhibit these functions, so strategies are needed to remove these xenobiotics from contaminated soils. In this regard, bioremediation is becoming one of the most commonly used techniques, with a large number of bioremediation methodologies being used satisfactorily [47,48,49]. Some of the interesting technologies for the removal of these contaminants in soils are biostimulation and/or bioenhancement provided during the application of organic amendments to contaminated soils; these bioremediation strategies have proven to be effective under certain conditions [50,51,52,53,54]. In addition, they are sustainable, low-cost, non-invasive, and can be carried out in situ. Some ways in which the pesticide concentration in contaminated soils has been reduced have been through the application of compost [55,56,57,58,59] or manure [60].

For all the above reasons, the objectives of this study were: (i) to compare and evaluate the effectiveness of the QuEChERS and QuEChERSER extraction methods on 13 pesticides, which represent a wide range of functional groups and polarities, in loamy-clayed soil; (ii) to determine the persistence of the herbicides PDMT and OXF and the fungicide TFXT in the selected soil using a microcosm test under laboratory conditions; and (iii) to evaluate the effect of the application of biowaste compost and cow manure on the natural persistence in the soil of the three selected pesticides.

## 2. Materials and Methods

### 2.1. Chemicals, Reagents, Soil, and Organic Amendments

**Figure 1:**
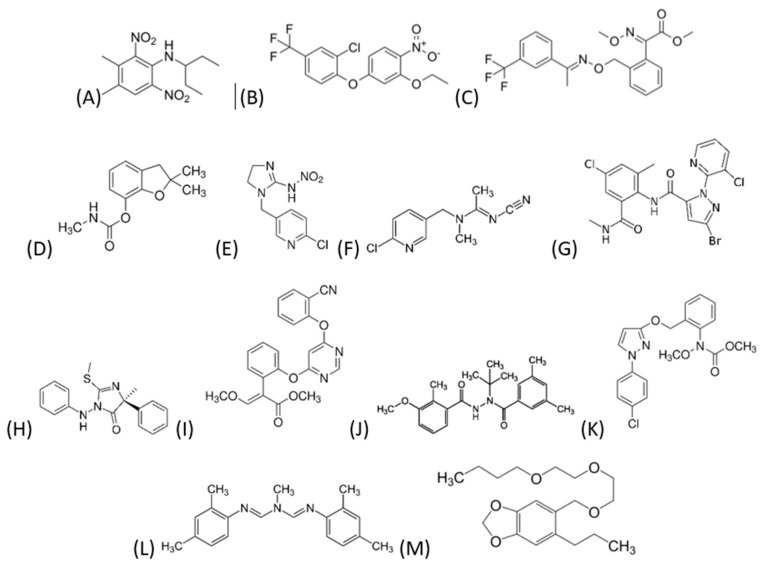
Chemical structure of pesticides studied: (**A**) PDMT, (**B**) OXF, (**C**) TFXT, (**D**) CB-30H, (**E**) IMDCPD, (**F**) ACMP, (**G**) CHLMP, (**H**) FNMD, (**I**) AZXB, (**J**) MTXFZD, (**K**) PCTB, (**L**) AMTZ, and (**M**) PIP-BTX.

Pure standards (>99%) from Dr. Ehrenstofer (Augsburg, Germany) were used. The following were used: methanol (MeOH) and acetonitrile (ACN) of LC-MS grade, Scharlab S.L. (Barcelona, Spain); ultrapure water HPLC, Milli-Q grade (Millipore, Bedford, MA, USA); formic acid (>98%, ACS reagent grade) and ammonium formate (>99%, LiChropur LC-MS grade) from Sigma-Aldrich (Darmstadt, Germany); QuEChERS EN ExtraBond extractive kit and dispersive kit (Scharlab, Barcelona, Spain); and pesticide mixture 301 × 10^5^ µg L^−1^ in can from LGC Standards Ltd., reference DRE-GA09000301AL, Dr. Ehrenstorfer (Augsburg, Germany). The following commercial products were used: Scorpio from Bayer (Leverkusen, Germany), Goal from Dow AgroSciences (Indianapolis, IN, USA), and StompLE from BASF (Ludwigshafen, Germany), which contained 330 g L^−1^ of PDMT, 240 g L^−1^ of OXF, and 500 g kg^−1^ of TFXT, respectively. Stock solutions of 1000 µg ml^−1^ were prepared by dissolving a sufficient amount in ACN and stored in amber glass bottles at a maximum temperature of 4 °C. The pesticides studied were: carbofuran-3OH (CB-3OH), imidacloprid (IMDCPD), acetamiprid (ACMP), chlorantraniliprole (CHLMP), fenamidone (FNMD), azoxystrobin (AZXB), methoxyfenozide (MTXFZD), pyraclostrobin (PCTB), amitraz (AMTZ), piperonyl-butoxide (PIP-BTX), PDMT, OXF, and TFXT. Figure 1 shows the chemical structure of each one. Their water solubility, Log K_ow_, and vapor pressure are shown in Table A1. The ones that are more soluble are ACMP and IMDCP. The most lipophilic are AMTZ and PDMT, and the most volatile are IMDCP and AMTZ. For quality control during ultra-high-performance liquid chromatography–tandem mass spectrometry (UHPLC-MS/MS) analysis, ^13^C-phenacetin was used as an internal standard, prepared at a concentration of 0.7 ng μL^−1^ in water. A mixture of the 13 pesticides containing 10 µg mL^−1^ was prepared. The standards of the prepared calibration curve contained the following concentrations: 0, 10, 20, 50, 100, and 200 ng mL^−1^ in the mobile phase. It is important to note that this mixture of compounds is representative of a variety of functional groups.

The soil sample used in the experiment corresponds to a Bt horizon of a calcium Luvisol. This soil was taken from the Mondúver mountain range (La Safor, Valencia, Spain). The sample was duly taken using the procedures described in the FAO Soil Description Guide [61]. The soil coordinates are: 39°02′24.3″ N 0°21′14.5″ W. The pre-treatment of the samples was conducted according to the procedure described by the Spanish Standard UNE-EN 16179 [62], which provides guidance for sample preparation of sludge, treated biowaste, and soil. Moisture content was determined by gravimetry following the Spanish Standard UNE-EN 16586 [63]. The pH was measured in a 1:5 (*v*/*v*) soil–water suspension according to the Spanish Standard UNE-EN 10390 [64]. Electrical conductivity (EC) was determined in the aqueous extract in a 1:5 (*w*/*v*) ratio using a Crison conductometer, following the Spanish Standard UNE-EN 77308 [65]. Granulometric analysis was performed using the Bouyoucos densimeter method as described by the Colombian Technical Standard NTC 6299 [66]. Carbonate content was determined by gas volumetry with a Scheibler apparatus, according to the Spanish Standard UNE-EN 10693 [67]. Oxidizable organic carbon was determined following the potassium permanganate oxidation method described in UNE 103204 [68], and total organic matter content was calculated by calcination according to the Spanish Standard UNE-EN 13039 [69].

Two organic amendments were used: Class A compost (C), made from biowaste and pruning waste, and cow manure (E) frequently used in agriculture. Their fundamental properties are shown in Table 1.

### 2.2. Extraction Methods Used

As indicated above, this study compares two extraction methods, QuEChERS (Q) and QuEChERSER (QS), in order to investigate which is more effective. The method of extracting pesticides from soils has traditionally been Q [70]. This is a type of dispersive solid phase extraction (d-SPE) used for sample preparation consisting of an initial extraction phase with 10 g of sample and 10 mL of ACN mixed with MgSO_4_ and NaCl in a 50 mL Falcon tube. After vortexing for 1 min and centrifugation at 5000 rpm for 5 min, the solid dispersive phase is performed, where 1 mL of the supernatant from the previous step is transferred to another 15 mL Falcon tube with primary secondary amine (PSA) and anhydrous MgSO_4_. Then it was stirred again and centrifuged. The supernatant was filtered with a 0.45 µm pore size nylon filter and vitalized. Quantification of the compounds in the extracts was carried out by UHPLC-MS/MS [13].

In 2021, a new method, QS, was published. This method is faster and requires the use of less material [23]. It consists of adding 2 g of the sample to a 15 mL Falcon tube together with 10 mL of a 4/1 solution of ACN/H2O. After vortexing for 10 min, it was centrifuged at 3500 rpm for 5 min. Then, 204 µL of the supernatant was taken and transferred to a 2 mL Eppendorf centrifuge mini-tube, and 25 µL of an internal standard (^13^C-phenacetin at 0.7 ng µL in water^−1^) and 571 µL of ultrapure water were added. It was then centrifuged at 13,000 rpm for 5 min. Finally, 500 µL of the supernatant was placed in a vial for analysis by UHPLC-MS/Ms.

**Figure 2:**
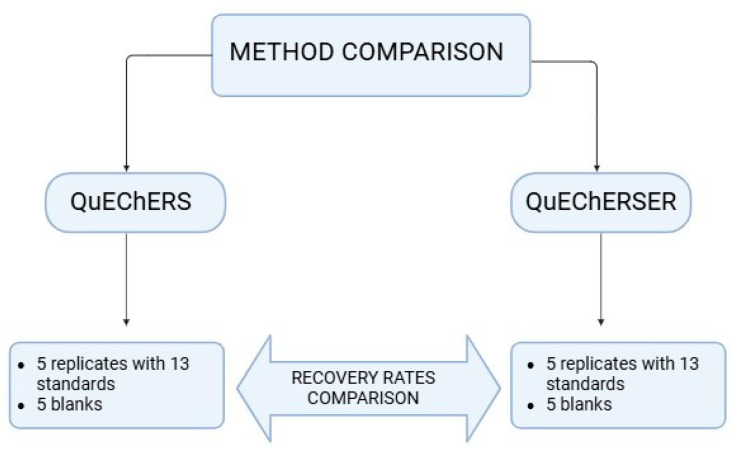
Schematic workflow comparing the QuEChERS (Q) and QuEChERSER (QS) extraction methods.

For extraction by method Q, 40 µL of a solution containing 10 µg mL^−1^ of the 13 pesticides to be tested was added to the soil, while for QS, 112 µL of this 10 µg mL^−1^ solution of the pure standards was added. Five replicates were made with their corresponding targets in order to validate both methods. The comparison process of both extraction procedures is shown schematically in Figure 2.

### 2.3. Quantification by UHPLC-MS/MS

The concentration of the analytes in all the extracts placed in vials was quantified by UHPLC-MS/MS liquid chromatography, with an ABSciex 6500 QTRAP mass spectrometer equipped with an electrospray ionization interface (ESI), using the following instrumental conditions: column C18, BEH waters 2.1 × 50 mm, 1.7 µm; temperature 35 °C; flow 0.35 mL min^−1^; and mobile phase, A: H2O (0.5% Formic acid) and B: ACN. The gradient was: %B: 5, 50, 60, 78, 88, 92, 100, 100, 5 with the following times respectively: 0, 1, 1.5, 2.5, 4, 5, 6, 7, and 8 min. For the quantification and confirmation of each pesticide, two product ions were monitored. For the identification and confirmation of the compounds, the relative response (ion ratio) was used together with their retention times (Table 2), as well as their quantification and confirmation transitions in a soil extract. The recovery tests were conducted on a control sample corresponding to the target soil type of the study at a concentration level of 28.6 µg L^−1^ of the pesticides analyzed. Linearity was evaluated by injecting soil extract in the concentration range between 0–100 µg L^−1^. The calibration curves were linear with 1/X adjustment in the concentration range with regression coefficients > 0.99, except for methoxyfenozide, which was 0.98. The limits of detection (LOD) and quantification (LOQ) were determined based on chromatographic peak area criteria following the US Food and Drug Administration’s Bioanalytical Method Validation Guidance (2018) [71]. The data are shown in Table 2.

### 2.4. Persistence Assay

**Figure 3:**
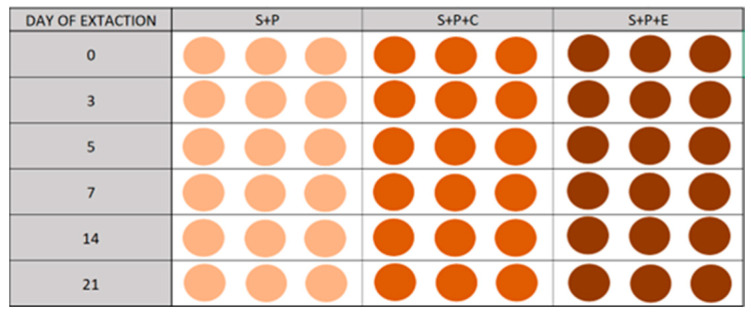
Experimental design of the microcosm persistence assay. Treatments: S+P (soil with pesticides); S+P+C (soil with pesticides and compost); S+P+E (soil with pesticides and cow manure).

A microcosm assay was conducted to study the persistence of PDMT, OXF, and TFX in the selected soil and the effect of the addition of the two organic amendments (C and E). Three treatments were performed in parallel: soil with pesticides (S+P), soil with pesticides and compost (S+P+C), and soil with pesticides and cow manure (S+P+E). Each experiment was conducted in triplicate. Samples were taken at 0, 3, 5, 7, 14, and 21 days (Figure 3). To confirm the results of the extraction method, both extraction methods tested were applied to the sample at the beginning of the experiment (t = 0) for the S+P treatment.

For the preparation of the experiment, 4.5 kg of soil were weighed and distributed in equal parts of 1.5 kg in three plastic trays. In the compost tray, 30 g of compost was added since the agronomic application dose is between 20 t ha^−1^ and 40 t ha^−1^. Furthermore, 21.5 g were added to the manure tray, taking into account that the agronomic application dose is 20 t ha^−1^. The soil was completely homogenized, and the organic amendments were applied to ensure that there was contact between the two. Of the commercial pesticide products, 10 times the manufacturer’s recommended application dose for rice cultivation was placed in each soil tray, simulating conditions of high contamination in the soil. For Scorpio (PDMT), this dose was 0.25 kg ha^−1^, for Goal (OXF) it was 1 L ha^−1^, and for StompLE (TFXT) it was 3 L ha^−1^. Thus, in our case, the dose to be applied was calculated based on the amount of soil, and it was obtained that 260 µg of Scorpio, 30 µL of StompLE, and 10 µL of Goal had to be applied. As the amount for Scorpio was so small, a solution of 100 mg L^−1^ of commercial product was made, and 2.6 mL of this solution was applied. These quantities of commercial products were diluted in 315 mL of Milli-Q water, which was distributed in each of the trays. This amount of water was used because it was the amount corresponding to 70% of the soil’s water holding capacity.

Each dose of pesticide was added to a spray bottle together with 315 mL of water and distributed over the soil surface in the tray while mixing, spraying, and washing the walls of the bottle with the different applications of water. Thus, the theoretical initial application dose of the pesticides per soil mass was 6600 µg kg^−1^ of PDMT, 1600 µg kg^−1^ of OXF, and 86.6 µg kg^−1^ of TFXT. Subsequently, the soil was distributed in sterile 100 mL containers. Each control point of the study was performed in triplicate. A total of 80 g of their respective mixture was placed in each. Each container was correctly labeled, indicating the experiment, day, and repetition. All containers were incubated with the unclosed lids in a dark chamber at room temperature (25 ± 2 °C) for the 21-day duration of the experiment. The only ones that were not incubated were those on day 0, which were directly frozen. For each day of extraction, only the 9 soil samples corresponding to that day were frozen to perform the extraction together. Every 2 to 3 days, both temperature and humidity were monitored by weight.

To interpret the dissipation of the applied pesticides, in each case, first-order regression models were fitted (Equation (1)). Pesticide dissipation in soil is often assumed to be a first-order reaction [72,73,74,75,76,77].

C = Co e^−kt^ (1)

where

- C is the concentration after a given time (t)
- Co is the initial concentration
- k is the dissipation constant

The half-life time was calculated with the regression equation using Equation (2):

t_1/2_ = Ln 0.5/k (2)

All calculations were performed using Microsoft Office Excel (version 2021 18.0). In order to evaluate the effect of the treatments on the degradation time of the pesticides, a one-factor ANOVA and Tukey’s Post Hoc test were performed. Previously, the homogeneity of the variances was checked with Levene’s statistic, and a test for the normality of the variables (Kolmogorov-Smirnov test) was performed. A t-test for equality of means was also performed, in which the Levene test was previously carried out, as well as the normality test to determine the differences between the recoveries in the extraction methods. The program used was IBM’s SPSS Statistics (version 28).

## 3. Results and Discussion

### 3.1. Soil Characteristics

Table 3 shows the results obtained in the analyses of pH, electrical conductivity, humidity, oxidizable organic carbon (COOx), organic matter (MO), and carbonates. The particle size analysis showed the following composition: 31% sand, 39% clay, and 30% silt. Thus, it is a loamy, basic soil, without salinity problems, with a moderate content of organic matter and it is slightly calcareous. This suggests that the soil has a good retention capacity for contaminants.

### 3.2. Comparison of Extraction Method

**Figure 4:**
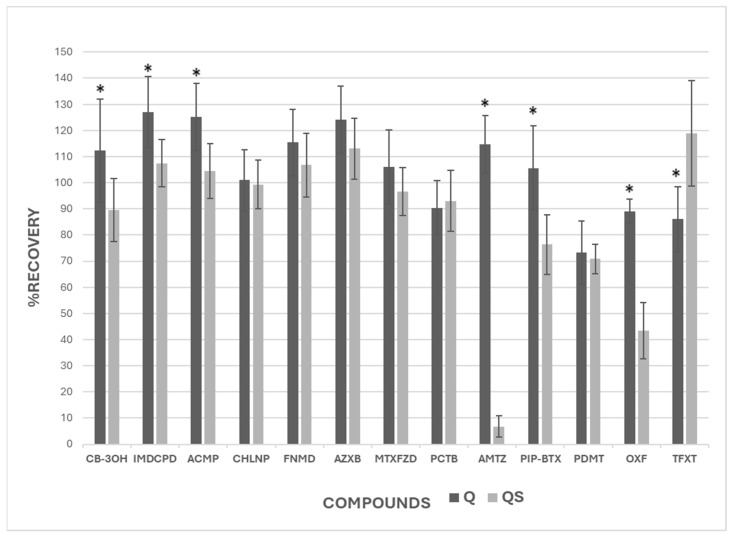
Recovery rates (%) of target pesticides using QuEChERS (Q) and QuEChERSER (QS) methods. Asterisks indicate significant differences between methods (mean equality *t*-test, *p* < 0.05).

After extracting and quantifying the 13 compounds added to the soil, similar recovery percentages were obtained for 6 of the 13 pesticides. Figure 4 shows the recovery of the compounds in each of the extraction methods and their standard deviation. The recovery data for each method, along with their standard deviations and coefficient of variation (CV), are shown in Table A2.

As can be seen, except for AMTZ and OXF, the recovery percentages of all pesticides analyzed are valid in both methods since they are within the accepted range of 70–120%, or 70–130% if their relative standard deviation (RSD) is ≤20 % [78]. The QS method was less satisfactory for extracting AMTZ and OXF, which may be due to the properties of the compounds or the interaction with soil constituents. The low recovery of AMTZ observed in the QS method can be explained by several factors. Although hydrolysis of amitraz is a relevant process contributing to its degradation [79,80,81], it may not be the only cause. It has been observed that amitraz tends to strongly interact with soil constituents, leading to its adsorption onto soil colloids, which hinders its extraction [82,83,84]. Additionally, in the QS method, the absence of salts such as MgSO_4_ and NaCl, used in the Q method, plays a significant role in the reduced recovery. These salts act as ‘salting out’ agents, facilitating the transfer of relatively polar organic compounds into the organic phase, which enhances their extraction. The lack of these agents in the QS method could be one of the reasons why amitraz is not extracted as effectively. Thus, besides hydrolysis, the interaction with soil components and the absence of salts in the extraction procedure significantly contribute to the low recovery observed. In the case of OXF, as well as AMTZ, it is a pesticide that is strongly retained in the organic matter of soils [85], with high K_ow_, resulting in high persistence [39,86]. The QS method, which does not use salts to extract compounds in any of its phases, may not be able to remove this pesticide from the soil organomineral complex, which would also explain the low recovery result, showing it does not work well for both AMTZ and OXF pesticides. If we individually compare the recoveries of both methods, it is observed that Q and QS recover soil compounds similarly. The mean of all valid Q recoveries is 105.4%, whereas QS has a mean recovery of 100.6%. In most pesticides, where Q appears to be a slightly better extractor, it tends to overestimate the recoveries above 100%, while QS generally yields values closer to the ideal 100%, reflecting a more accurate estimation. The results of the statistical analysis showed that there were significant differences between the extraction methods for 7 of the 13 pesticides.

**Figure 5:**
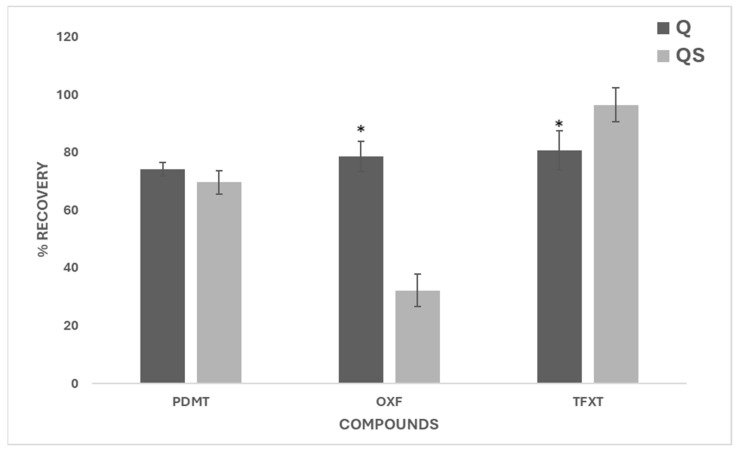
Initial recovery percentages (time = 0) of PDMT, OXF, and TFXT in the soil + pesticides (S+P) treatment. Asterisks denote significant differences between Q and QS methods (mean equality *t*-test, *p* < 0.05).

Furthermore, Figure 5 shows the results obtained for PDMT, OXF, and TFXT extracted by the two methods at the beginning of the experiment (t = 0) for the soil persistence study. Statistical analysis demonstrated significant differences between both methods for OXF and TFXT, while no significant difference was obtained for PDMT. The Q method was more effective at extracting OXF, while QS was more effective at extracting TFXT. These results confirmed those obtained in the previous trial.

Although the recovery results were not the best for the OXF in QS, the main reasons for selecting QS as extraction method, and PDMT, OXF and TFX for the persistence experiment were: (i) they are widely used in soils dedicated to intensive agriculture in our area; (ii) we believed that greater or lesser recovery would have little effect on the dissipation results; (iii) moreover, our main interest was to obtain information about what occurred in the persistence trials using the QS extraction method, in order to confirm, or not, the results obtained in the extraction assay, and (iv) QS is faster and robust; then, as indicated by Lehotay (2022) [87], QS method is substantially better than Q for nonpolar analytes in matrices like soils. In addition, its use enables us to improve the knowledge we have about this method for the extraction of these three pesticides in soils.

### 3.3. Pesticide Persistence in Soil and Effect of Organic Amendment Applications

Table 4 shows the concentration of the pesticide products at each sampling time for the three treatments performed, and Table 5 shows the parameters of the first-order degradation kinetics equations, as well as the half-life times and the ANOVA result for each compound and treatment applied. The three pesticides showed different behaviors. Except in the OXF control, in all other cases, it can be observed that the concentration of the pesticides decreased after 21 days. The concentration of PDMT dissipated moderately; OXF also dissipated moderately, except in the control, in which it did not dissipate. TFXT dissipated much faster in all cases. In addition, it was observed that the application of organic amendments (S+P+C and S+P+E) also favored the dissipation of pesticides in all cases with respect to the control (S+P) (Table 4).

Regarding PDMT, in the S+P treatment, no measurable dissipation was observed. In contrast, after compost amendment, the reduction was 35.2%, and with manure it was 28.9%. In this sense, Garci-Valcarcel et al. (2003) [88] demonstrated that persistence is greater in soils that have not been treated with organic amendments, which is explained by the fact that PDMT interacts with the soluble organic matter provided by organic amendments, so it is more bioavailable for degradation. Another study by Tsiropoulos & Miliadis (1998) [89] showed that the half-life time of PDMT in soil increased slightly with dose. When the minimum recommended dose was applied, the half-life time was 37 days, whereas when the maximum recommended dose was applied, it was 39 days. In our case, 10 times the recommended average dose was applied, so the half-life time increased to 63 days in the control. Other authors [90] have shown that PDMT can persist in the soil for up to 90 days, coinciding with the wheat harvest period; however, it was observed that persistence can be influenced by cultivation practices, soil temperature and humidity, as well as soil type. Although a decrease in herbicide concentration was observed in amended soils at the end of the experiment (Table 4), no significant differences in half-life were found between treatments (Table 5).

OXF showed high variability at the beginning of the experiment on tree treatments, which may be due to the spraying, homogenization, and sampling process. This variability decreased due to the moistening and homogenization performed in each sampling. Then, OXF did not reduce its concentration in the S+P treatment during the experiment. Compost reduced its initial concentration by 23.3%, while manure did so by 25.8%. These results are similar to those obtained in other studies [86,91,92], which show that the low concentration of OXF in the soil is related to its high content of organic matter after the application of organic amendments, which favors microbiological activity, thus accelerating its biodegradation and decreasing its persistence. Furthermore, the study conducted by Mohamed et al. (2011) [93] shows that degradation in soil without the addition of organic matter is much lower at 28 °C than at 40 °C, so it is highly influenced by temperature. The temperature at which this experiment was carried out was around 25 °C, so degradation in the control was practically zero. This study also shows that the application of fertilizers increased the degradation of OXF. On the other hand, other studies [94,95] show that OXF degrades more poorly in clay soils. Kumbhar & Mukesh (2017) [94] observed that the concentration of this compound in clay soils was reduced by only 9% after 20 days. This experiment was conducted on loamy-clayed soil, so the high persistence of the pesticide in the control soil can also be explained by this aspect. Mantzos et al. (2014) [96] obtained that OXF has low mobility in the soil and does not leach, as it was detected in very low concentrations below 10 cm depth. This shows that its potential to contaminate adjacent waters is very low and that where it is applied is where it persists. The ANOVA results showed significant differences between the treatments, indicating that the application of organic amendments favors the dissipation of this herbicide in the soil, although the effect of compost or manure application was not significant between them. The half-lives were 53 days in compost and 58 days in manure (Table 5).

Regarding TFXT, the results show that there is a constant and noticeable decrease in its concentration in all cases. After 15 days, it had practically dissipated in the S+P treatment and the amended soils. This fungicide is undoubtedly a case of the pesticide dissipating the fastest in the soil, both after the application of organic amendments and without their application. At the end of the experiment, the concentration in S+P was reduced by 91.4%, while that of S+P+C was 94.2%, and in S+P+E was 97%. Quite a few studies have demonstrated the degradation of TFXT in soil, as well as in soil and crop mixtures susceptible to fungal diseases [44,97,98,99]. In some cases, it has been observed to decompose rapidly in soils, and its residues have been shown to dissipate below the detection limit in 15 days. Additionally, its half-life time was 3.48 days when its application dose is doubled in grape leaves [44]. Its half-life times vary depending on the dose of application, but they show the pattern that an increase in dose leads to an increase in half-life time. They all lie between the maximum value of 6.2 days and the minimum of 1.58 days. The half-life time obtained in our experiment was 5.2 days in the S+P and in S+P+C soil and 4.5 days in the S+P+E treatment, consistent with the results previously discussed. Another study conducted by Banerjee et al. (2006) [100] showed that TFXT degradation is independent of latitude and that it is effectively degraded in different soils in different geographical regions. In addition, it confirms that it is strongly adsorbed in the soils studied. We have not found studies demonstrating the effectiveness of the application of organic amendments in the degradation of TFXT; however, other studies with other strobilurins have shown that the application of organic amendments improves its degradation AZXB, PCTB and TFXT, all share the characteristic methoxyacrylate pharmacophore and a highly lipophilic aromatic core—features that promote strong soil adsorption and render them susceptible to microbial hydrolysis [101,102]. Although bibliographic data on TFXT is limited, these structural and physicochemical similarities among the three strobilurins suggest that TFXT may respond to organic amendments in much the same way as AZXB and PCTB. Thus, it has been observed that the degradation of AZXB showed a significant decrease in its half-life time after compost application [103]. Other authors [74] observed that the half-life times of AZXB decreased in soils with a higher content of organic matter, nitrogen, and biomass carbon, as well as with higher respiration rates, which supports the role of biological activity in the degradation of this type of fungicide. Zhang et al. (2021) [104] also observed that the application of biochar to soils reduced the inhibitory impact of PCTB on soil microbiota, as well as the pesticide concentration itself in the soil. In our case, the statistical analysis showed that the application of a compost is not significant for the half-life time of TFXT in soil. However, the application of cow manure did significantly reduce the average lifetime. Either way, TFXT’s half-life time was very short in all cases.

Finally, it should be noted that the application of organic amendments led to a decrease in half-life times in all cases, except for TFXT after compost application. In this case, the half-life remained as short as in the control (5.2 days). Compost decreased, to a greater extent, the half-life time with respect to manure (33 days with respect to 50 days for PDMT and 53–58 days for OXF) (Table 5), which is equivalent to reductions of 45% and 77% respectively. For TFXT, it remained the same. With the application of cow manure, the half-life time was reduced by 20% for PDMT, 74.9% for OXF, and 13.5% for TFXT.

## 4. Conclusions

In this study, the QS mega-method was compared with the classical Q method for the analysis of 13 pesticides in a Mediterranean calcic Luvisol. Additionally, the persistence of three of these pesticides (PDMT, OXF, and TFXT) was evaluated in this soil under three conditions: without amendment (S+P), with biowaste compost (S+P+C), and with cow manure (S+P+E).

The application of both extraction methods showed that, in most cases, Q and QS recovered a significant proportion of the target compounds, although the efficiency varied depending on the pesticide. Notably, the QS method showed low recoveries for AMTZ, and OXF, showing that it is not a suitable method for their extraction. These results suggest that further research is needed to clarify under which conditions and for which compounds the QS method performs reliably, in order to validate its broader application across different soil types.

Following the evaluation of the persistence of PDMT, OXF, and TFTX in the selected soil and the effect of the application of two organic amendments (biowaste compost and cow manure), it was found that the herbicide OXF was the most persistent, while the fungicide TFXT showed very little persistence; the herbicide PDMT showed an intermediate behavior. PMDT and OXF were persistent in the non-amended soil. The soil persistence of the three pesticides decreased after the application of compost or cow manure. The half-life times obtained in the S+P+C and S+P+E treatments were: 33 d and 50 d for the herbicide PDMT, and 53 d and 58 d for OXF. For the ungicide TFXT, the half-life times were 5.2 d, 5.2 d, and 4.5 d for S+P, S+P+C, and S+P+E, respectively. The application of the two organic amendments, under laboratory conditions, significantly decreased the half-life time of PMDT, OXF, and TFXT. PDMT and OXF significantly reduced their half-life time after application of both compost and cow manure; the TXFT did so after the manure application.

Future studies might perform validation in different soils and evaluate the recovery of other pesticides. In addition, more studies are needed in order to determine whether the application of organic amendments facilitates the degradation of these pesticides and under what conditions they are more effective. It is also necessary to study the impact of these compounds on soil biodiversity and soil properties in order to promote more appropriate legislation and standards for the use of these substances, as well as stricter controls to avoid their excessive use and unnecessary risks.

## Figures and Tables

**Table 1:** Compost and manure characteristics.

	Compost	Manure
pH	8.74	9.07
EC (dS m^−1^)	5.20	11.22
OM (%)	78.55	82.99
OOC (%)	27.51	28.1
N (%)	2.40	2.60

EC: electrical conductivity. OM: organic matter. OOC: oxidizable organic carbon.

**Table 2:** Validation parameters of pesticides in soil samples.

Compound	Linearity (r2)	RT (min)	Q1 (*m*/*z*)/Q3 (*m*/*z*)	LOD (µg L^−1^)	LOQ (µg L^−1^)
CB-3OH	0.999	2.52	238/163.2	2	6.38
IMDCPD	0.999	2.57	256.1/209	2	5.68
ACMP	0.999	2.63	223/125.9	2	5.40
CHLNP	0.997	3.61	484.1/453.1	2	5.44
FNMD	0.999	3.88	312.1/92.3	2	5.43
AZXB	0.997	3.82	404.2/329	1	2.70
MTXFZD	0.980	3.98	369.3/149.1	2	5.89
PCTB	0.996	4.43	388.1/163	2	5.43
AMTZ	0.999	4.61	294.3/163.2	2	5.41
PIP-BTX	0.992	4.84	356.2/177.1	2	6.20
PDMT	0.999	5.05	282.1/212	2	4.50
OXF	0.999	4.88	362.1/316.9	2	4.56
TFXT	0.999	4.63	409.1/186.1	2	6.46

RT: retention time. Q1 = precursor ion. Q3 = product ion. LOD: limit of detection. LOQ: limit of quantification. CB-3OH: carbofuran-3OH. IMDCPD: imidacloprid. ACMP: acetamiprid. CHLNP: chlorantraniliprole. FNMD: fenamidone. AZXB: azoxystrobin. MTXFZD: methoxyfenozide. PCTB: pyraclostrobin. AMTZ: amitraz. PIP-BTX: piperonyl-butoxide. PDMT: pendimethalin. OXF: Oxyfluorfen. TFXT: trifloxystrobin.

**Table 3:** Soil core properties.

	pH	EC (dS m^−1^)	OOC (%)	OM (%)	Carbonates (%)
Mean	8.03	0.129	1.92	3.30	6.94
SD	0.21	0.001	0.04	0.08	0.36
CV(%)	2.60	0.775	2.34	2.42	5.18

EC: electrical conductivity. OOC: oxidizable organic carbon. OM: organic matter.

**Table 4:** Pesticide concentration (µg kg^−1^) at each sampling date for the three treatments performed (S+P, soil with pesticides; S+P+C, soil + pesticides + compost addition; and S+P+E, soil + pesticides + manure addition). Data represent mean ± standard deviation, with dissipation percentages (%D) calculated from day 0 to day 21.

	PDMT			OXF			TFXT		
Day	S+P	S+P+C	S+P+E	S+P	S+P+C	S+P+E	S+P	S+P+C	S+P+E
Theoretical initial concentration	6600			1600			86.6		
0	4524 ± 640	4590 ± 809	4273 ± 966	576 ± 52	691 ± 75	1023 ± 100	80 ± 4	70 ± 3	86 ± 12
3	3475 ± 621	4100 ± 536	3640 ± 296	959 ± 98	728 ± 124	869 ± 18	87 ± 13	35 ± 6	32 ± 3
5	3251 ± 508	3568 ± 584	3650 ± 434	739 ± 117	629 ± 100	796 ± 130	47 ± 8	20 ± 2	15 ± 3
7	4041 ± 501	3409 ± 573	3145 ± 523	624 ± 114	612 ± 120	887 ± 72	18 ± 5	13 ± 1	14 ± 3
14	3212 ± 144	2917 ± 157	3193 ± 228	605 ± 154	604 ± 69	769 ± 51	8 ± 3	5 ± 1	5 ± 1
21	3319 ± 460	2974 ± 578	3034 ± 474	735 ± 99	530 ± 67	759 ± 145	6 ± 0	4 ± 0	2 ± 0
%D	ND	35	29	ND	23	25	91	94	97

ND, No dissipation.

**Table 5:** First-order kinetics parameters for each treatment and its half-life.

Treatment		C_0_ (µg kg^−1^)	k	t_1/2_ (day)	R^2^
Pendimethalin	S+P	3938.9	−0.011	-	0.3638
	S+P+C	4206.6	−0.021	33.0 a	0.8220
	S+P+E	3888.3	−0.014	49.5 a	0.7057
Oxyfluorfen	S+P	701.3	−0.001	-	0.0074
	S+P+C	700.8	−0.013	53.0 a	0.7949
	S+P+E	931.4	−0.012	58.0 a	0.6331
Trifloxystrobin	S+P	81.9	−0.134	5.2 a	0.8256
	S+P+C	47.9	−0.133	5.2 a	0.9208
	S+P+E	52.6	−0.154	4.5 b	0.8837

For each analyzed compound, the same letter between treatments indicates no significant differences (Tukey, *p* < 0.05).

## Data Availability

The authors confirm that the data supporting the findings of this study are available within the article.

## Appendix A

**Table A1:** Physicochemical properties of target pesticides.

Compound	Water Solubility (mg/L) (20 °C)	Log Kow (21°)	VP (mmHg) (25 °C)
CB-3OH	NA	NA	NA
IMDCPD	610	0.570	4.4 × 10^−5^
ACMP	4250	0.8	NA
CHLNP	1	2.76	NA
FNMD	NA	NA	NA
AZXB	6	2.50	8.3 × 10^−13^
MTXFZD	3.3	3.7	NA
PCTB	1.9	3.99	1.95 × 10^−10^
AMTZ	1	5.5	2 × 10^−6^
PIP-BTX	14.3	4.75	2.6 × 10^−7^
PDMT	0.33	5.20	2.4 × 10^−6^
OXF	0.116	4.73	2.1 × 10^−7^
TFXT	0.61	4.5	2.5 × 10^−8^

NA: not available. Source: National library of medicine. PubChem.US 2024. [105].

**Table A2:** Recovery rates (mean), standard deviation (SD), and relative standard deviation (RSD) of target pesticides using QuEChERS (Q) and QuEChERSER (QS) methods.

	Q			QS		
Compound	Mean	SD	RSD	Mean	SD	RSD
CB-3OH	112.2	19.8	17.7	89.6	12.0	13.4
IMDCPD	126.9	13.6	10.7	107.5	9.1	8.4
ACMP	125.2	12.8	10.2	104.5	10.5	10.1
CHLNP	100.9	11.5	11.4	99.3	9.2	9.3
FNMD	115.5	12.7	11.0	106.7	12.1	11.4
AZXB	124.2	12.8	10.3	113.1	11.7	10.3
MTXFZD	106.1	14.2	13.4	96.6	9.2	9.5
PCTB	90.4	10.4	11.5	93.1	11.6	12.4
AMTZ	114.7	11.0	9.6	6.8	4.1	60.4
PIP-BTX	105.6	16.2	15.3	76.3	11.5	15.0
PDMT	73.3	12.2	16.6	70.8	5.5	7.8
OXF	89.1	4.5	5.1	43.5	10.8	24.9
TFXT	86.0	12.4	14.4	118.9	20.2	16.9
